# An Overview on the Primary Factors That Contribute to Non-Allergic Asthma in Children

**DOI:** 10.3390/jcm11216567

**Published:** 2022-11-05

**Authors:** Angela Klain, Giulio Dinardo, Alessandra Salvatori, Cristiana Indolfi, Marcella Contieri, Giulia Brindisi, Fabio Decimo, Anna Maria Zicari, Michele Miraglia del Giudice

**Affiliations:** 1 Department of Woman, Child and General and Specialized Surgery, University of Campania “Luigi Vanvitelli”, 80138 Naples, Italy; 2 Department of Pediatrics, Sapienza University of Rome, 00161 Rome, Italy

**Keywords:** non-allergic asthma, children, exercise-induced asthma, premenstrual asthma, viral infections, pollution

## Abstract

The prevalence of non-allergic asthma in childhood is low, peaking in late adulthood. It is triggered by factors other than allergens, like cold and dry air, respiratory infections, hormonal changes, smoke and air pollution. In the literature, there are few studies that describe non-allergic asthma in pediatric age. Even though it is a less common disorder in kids, it is crucial to identify the causes in order to keep asthma under control, particularly in patients not responding to conventional treatments. In this review, we discuss non-IgE-mediated forms of asthma, collecting the latest research on etiopathogenesis and treatment.

## 1. Introduction

**Figure 1:**
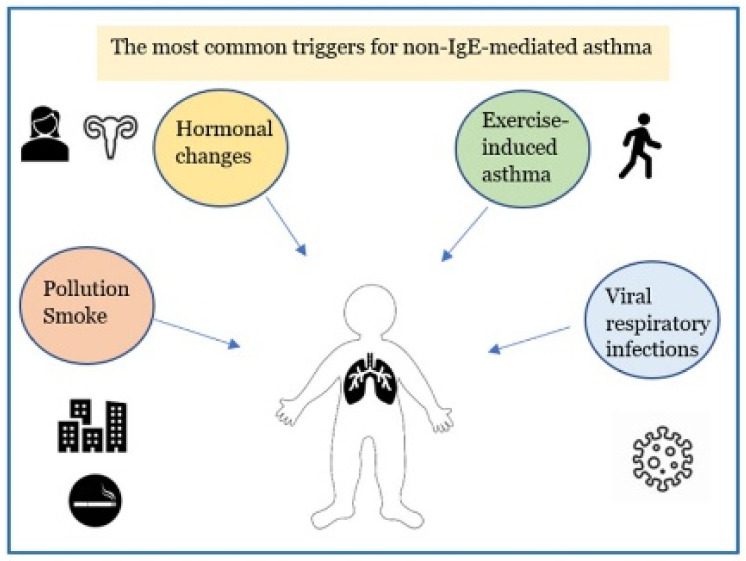
The major factors leading to non-allergic asthmatic exacerbations in children.

Asthma is the most common chronic respiratory inflammatory disease in children [1,2,3,4]. Pediatric asthma includes infantile asthma (from 6 months to 2 years of life), asthma in pre-school and school-aged children up to the adolescent period [5]. It is described as a heterogeneous disease that includes different clinical conditions for etiopathogenesis, biological bases, clinical manifestations, course in time and response to therapy [6,7,8,9,10]. More recently, it has become possible to distinguish between two distinct “endotypes” (which identify the biomolecular bases connected to particular clinical phenotypes): the high expression T helper (Th) 2 model corresponds to allergic asthma which accounts for 50–80% of all cases [11,12,13,14] and the low expression Th2 model includes the non-allergic forms of asthma. Non–allergic asthma (NAA), previously identified as intrinsic asthma, is less frequent (10–30% of asthmatic patients [15]) and compared with the low Th2 expression model it has a later onset than that in allergic asthma, with a female predominance. The symptoms of NAA are caused by triggers other than allergens, such as exercise, pollutant inhalation, sexual hormonal changes, and virus infections [Figure 1]. The biomolecular mechanism of the high expression Th2 model is supported by the inflammatory cascade that sees the activation of Th2 lymphocytes with the activation of type 2 innate lymphoid cells (ILC2), expression of particular cytokines, such as interleukin IL-4, IL-13, IL-5, and GM-CSF (Granulocyte-Macrophage Colony-Stimulating Factor), resulting in the recruitment, proliferation and activation of eosinophils. The allergic reaction is characterized by three phases: sensitization to an allergen, early phase response and late-phase response. Initially, the allergen is digested into tiny peptide fragments by an antigen-presenting cell. The major histocompatibility complex molecules and the peptide fragments are presented on the surface of the antigen-presenting cells to Th2 cells. The Th2-cells release cytokine, IL-5, IL-4 and IL-13, which induce the development of allergen-specific IgE antibodies by B cells that have recognized the same allergen. IgEs enters the circulation and adhere to the membrane of mast cells, which have the task of identifying the allergen when it comes into contact with the organism for the second time (immunological memory). In the second phase, mast cells are activated by IgE-binding epitopes, and release mediators such as histamine, prostaglandins, leukotrienes, which cause dilatation of blood vessels, constriction of the bronchioles, increased mucus secretion, and other allergy symptoms caused by inflammatory mediators binding to receptors on target cells. Most people start to suffer “early phase” allergy symptoms, including sneezing, wheezing, itchy eyes, runny nose, cough, and hives, after a few minutes of being exposed to the allergen. Typically, the “late-phase” starts 2–6 h after exposure to the allergen. Numerous additional inflammatory cells, including as eosinophils, basophils, neutrophils, and lymphocytes, are drawn to the area by the activated mast cells, resulting in tissue damage [16]. On the other hand, the low expression Th2 model, where innate/acquired immune processes play a major role, results in the activation of mediators and various cellular populations, such as Th17, less responsive to inhaled corticosteroids [16,17,18,19]. The mechanisms contributing to the low expression Th2 model in asthmatic patients are less clear: dysregulated neutrophil-mediated immune responses due to respiratory infections [20] or defects in resolution of inflammation [21], and the activation of the IL-17-dependent pathway [22,23] have been proposed as possible mechanisms involved. In mucosa and bronchoalveolar lavage fluid of individuals with NAA, there is a higher expression of the chemokine RANTES (Regulated on Activation, Normal T cell Expressed and Secreted) and higher levels of neutrophil-derived mediators (leukotriene B4 (LTB4), GM-CSF, tumor necrosis factor a (TNFa), IL-17A, IL-8, elastase, or metalloproteinase 9) [24,25]. During asthma exacerbations the cytokines IL-8 and IL-17A prolong the life span of the neutrophil and modulates Th2 and innate immune responses. Additionally, because of their longer lifespan, neutrophils can move from tissues to the bloodstream or lymph nodes to influence adaptive immune responses [26]. A lack of allergy history, negative skin prick or in vitro-specific immunoglobulin E (IgE) tests to a panel of local and perennial allergens, total serum IgE levels typically normal or low (<150 IU/mL) [27], raise suspicions of NAA.

### 1.1. Exercise-Induced Asthma (EIA)

The first crucial distinction to be made is between exercise-induced asthma (EIA) and exercise-induced bronchoconstriction (EIB). Bronchial hyperactivity and persistent inflammation are the hallmarks of the genuine pathology known as EIA; on the contrary, the temporary airway restriction known as EIB may also be present in non-asthmatic patients [28]. EIB is presented in 52.5% of children with asthma [29,30] and it occurs in 8.6–12% of healthy children aged 7–17 years [31], EIA is triggered by cold and dry air during exercise, causing dehydration of the airways mucosa, resulting in increased osmolarity, contraction of bronchial smooth muscle, and an influx of eosinophils and mast cells that release inflammatory mediators (leukotrienes, histamine, IL-8, tryptase, and prostaglandins) [32]. These signalling molecules lead to an increase in smooth muscle contraction, mucus formation, microvascular permeability, and sensory nerve activation of the airways, which is the main cause of bronchoconstriction and airway oedema [28,33,34]. Recent studies report correlations of bronchospasm with eosinophils [35], eosinophil cationic protein (ECP) [36], lipoxin A4 [37], phospholipase A2 [38], and endothelin-1 [39,40]. Symptoms often begin to manifest 5–8 min after the beginning of continuous high-intensity exercise, or 2–5 min of heavy exercise [41,42]. EIA can be diagnosed with spirometry after an exercise challenge test [43]. A difference of more than 10% between the lowest FEV1 value obtained during 30 min of exercise and the pre-exercise FEV1 value is diagnostic of EIA. Higher values for a percent fall in FEV1 (i.e., 15 and 13.2%) have been recommended for diagnosing EIA in children [44]. The American Thoracic Society (ATS) advises using a short-acting β2-agonist (SABA) 5–20 min prior to exercise in patients with EIB and EIA. Before exercise, daily inhaled corticosteroids, daily leukotriene receptor antagonists, or mast cell stabilizing drugs should be used for patients who still experience the symptoms despite the administration of SABA [33,45,46,47]. Children with EIA frequently have significant limitations to sports and physical activity. However, regular exercising can be safe for kids as long as the proper precautions are taken: avoiding or minimizing activity in highly polluted regions, exposure to cold air, performing a pre-exercise warm-up, regular physical activity, and adequate control of asthma [48,49,50]. Several studies have shown a protective benefit of HME (heat and moisture exchanger) face mask in counteracting EIB, as indicated by a slowed post-exercise decline in FEV [51,52]. However, in childhood this preventive strategy is not much feasible, because children, in particular those with asthma-related conditions, are unlikely to wear the masks.

### 1.2. Virus-Associated Asthma

Viral respiratory infections represent one of the most common triggers for asthma exacerbations and wheezing in newborns and children [53] and they have also been clearly implicated in the development of asthma [54]. Human rhinovirus (RV), particularly subtypes A and C, is the most frequent viral pathogen, causing around 60% of virus-associated asthma exacerbations in school-age patients [55]. Other respiratory viruses, as respiratory syncytial virus (RSV), may cause exacerbations and/or induction of asthma. RSV is the major one, followed by coronaviruses, parainfluenza viruses, human metapneumoviruses, and adenoviruses [56]. In particular, RSV infection is more frequently linked to the induction of asthma [57]. The mechanisms that lead to asthma exacerbations or virus-induced asthma are poorly understood, emerging evidence points out the theory of the presence of a lack in antiviral immunity and a loss of epithelial barrier integrity in patients with severe asthma [58], while the possibility for an infection to cause a disease depends on virus’ characteristics (i.e., type, virulence), host (i.e., age, comorbidity, genetic susceptibility), and environmental factors (i.e., season). It is known that all respiratory viruses have the capacity to enter and replicate within ciliated and non-ciliated respiratory epithelial cells, leading to necrosis, loss of cilia, ciliostasis, and impairment of muco-ciliary clearance [59]. RV and RSV are the principal cause of bronchiolitis, a lower respiratory tract infection, in children under 2 years, resulting in the development of wheezing and in important breathing difficulties, requiring often hospitalization [60]. According to Corne et al., children with asthma are more susceptible to viral infections and they are associated with more severe clinical manifestations, with a worsened airflow obstruction and a lower mean forced expiratory volume (FEV1), and increased risk of hospitalization [61].

Many studies have investigated the effect of the RV infection on respiratory cells, highlighting the capacity to determine an impaired respiratory epithelial function, causing the production and the release of proinflammatory mediators, such as IL-1, IL-6, chemokine C-X-C motif ligand 10 (CXCL10)/interferon-inducible protein 10 (IP-10), chemokine C-C motif ligand 5 (CCL5)/RANTES, and CXCL8/IL-8, which are critical for the start of neutrophilic (CXCL8/IL-8), lymphocytic (CXCL10/IP-10), and eosinophilic (CCL5/RANTES) inflammation, airway remodeling, and antiviral immune responses [62,63]. Message SD et al. investigated the role of Th1 and Th2 cytokines and IL-10 in a RV-induced asthma exacerbation model, based on the fact that asthma is associated with augmented Th2 immune responses and impaired IL-10 regulatory responses. Th2 cytokines IL-4, IL-5, and IL-13 were correlated with more severe virus-induced asthma symptoms; whereas IFN-γ (Th1 cytokines) and IL-10 were associated with minor symptoms and conditions (i.e., lower viral load). They demonstrated that in asthma, the Th1 cytokines and IL-10, which are produced to a lesser degree, were associated with protection from exacerbation, whereas Th2 cytokines, which on the other side are increased, were associated with increased disease severity. These observations provide convincing evidence supporting an important role for RV-induced lower airway inflammation in precipitating asthma exacerbations [64]. Legg J et al. demonstrated the presence of a deficient Th1 and an augmented Th2 immune response in children with RSV-induced bronchiolitis [65]. Moreover, Morano M et al. proved that children with an increased Th2 immune response during RSV-infection were at higher risk of wheezing during follow-up [66]. Several therapeutic strategies succeed in modifying the course of virus-induced asthma exacerbations, such as prevention of infection with vaccines or monoclonal antibodies; or treatment of infection with anti-inflammatory treatments, such as glucocorticosteroids; antiviral agents; and antibiotics. The treatment needs to be initiated as soon as possible during the infection to maximize the possibility of success. On the other hand, little is known about the possibility of successfully preventing a viral infection in order to minimize the risk of the development of asthma. During RSV season, it is possible to administer monthly to high-risk infants, including children younger than 24 months with chronic lung disease or preterm infants, Palivizumab, a humanized mAb directed against RSV F protein, which is able to reduce hospitalization in these children and seems also to have the capacity to reduce wheezing in children [57,67]. Another preventing option is represented by Ribavirin (1-ß-ribofuranosyl-12-3-carboximide), a synthetic nucleoside against DNA and RNA viruses, which can inhibit RSV replication, reducing complications such as pneumonia or bronchiolitis. For RV infection, several strategies have been tried or are still under investigation, such as prophylactic use of topical IFN-a, capsid binding agents, or soluble ICAM-1. They prevent viral uncoating or binding, preventing the infection [57], but in clinical practice their use is limited, because of their inefficacy; so they are recommended only after the onset of symptoms, to minimize them [68].

Patients with moderate and severe asthma have a widespread use of glucocorticoids because of their anti-inflammatory activity. Virus-induced exacerbations can occur also in well controlled ICS-treated patients, because of the presence of a specific virus-induced inflammation which is not controlled by stable ICS. In the study by Edwards et al. the association between salmeterol and fluticasone treatment suppressed the production of CXCL8, CCL5, and CXCL10 after RV infection in vitro [69]. Svevaki et al. showed that budesonide suppressed RV-mediated induction of proinflammatory cytokines (CCL5, CXCL8, IL-6, and CXCL10) and remodeling-associated factors (FGF and VEGF) in a concentration-dependent manner in vitro. Formoterol treatment also suppressed the production of CXCL8 and fibroblast growth factor (FGF) in vitro, but the association between the two drugs (budesonide and formoterol) showed a concentration-dependent, synergistic effect in suppression of RV-induced CCL5, CXCL8, CXCL19, and vascular-Endothelial Growth Factor (VEGF) [70]. All of these data support the use of glucocorticoids during asthma and their intensification during exacerbations.

In addition to viral-inducted or associated-asthma, few studies have investigated the association between bacterial infection and colonization with exacerbation and recurrent wheezing. At the moment, despite of what we have learnt about viral-infections and their causative role, it is still unclear if bacteria can be correlated in the same way to the development of asthma or to an augmented risk of exacerbations [71]. Surinder Kumar et al. studied 80 children aged 5–15 years, 50 with asthma and 30 without it for detection of *Mycoplasma pneumoniae* on nasopharyngeal aspirates and they affirmed that lower tract infections with *M. pneumoniae* are often associated with exacerbations of asthma in children [72]. Recently, through a systematic review and meta-analysis, Xiaoran Liu et al. have demonstrated a statistically significant association between *M. pneumonia* infection and an increased risk of any kind of childhood asthma [73]. However, we think that further well-designed and -controlled studies are required to evaluate this association and identify the underlying mechanisms.

### 1.3. Premenstrual Asthma

The function of sex hormones in the pathogenesis of asthma and its worsening has been the subject of much investigation for many years. Several studies show that fluctuations in sex hormones during puberty, menstruation, pregnancy and menopause affect the symptoms and severity of asthma [74,75,76]. It has been found that women have an increase in the prevalence of asthma during puberty compared to men, and that this discrepancy is caused by female sex hormones [77]. Overall, ovarian hormones elevate, and testosterone lowers airway inflammation in asthma, although the underlying mechanism is yet unknown. Premenstrual asthma (PMA), is a clinical situation that occurs in the luteal phase of the menstrual cycle, leading to a worsening of asthma symptoms. PMA has been estimated to affect up to 40% of asthmatic females. Some women may also experience systemic symptoms, including urticarial with or without angioedema, eczema, and, in severe cases, anaphylaxis. It seems that these manifestations are caused by a “progestogen hypersensitivity” due to progesterone surges during the luteal phase of the menstrual cycle [78].

**Figure 2:**
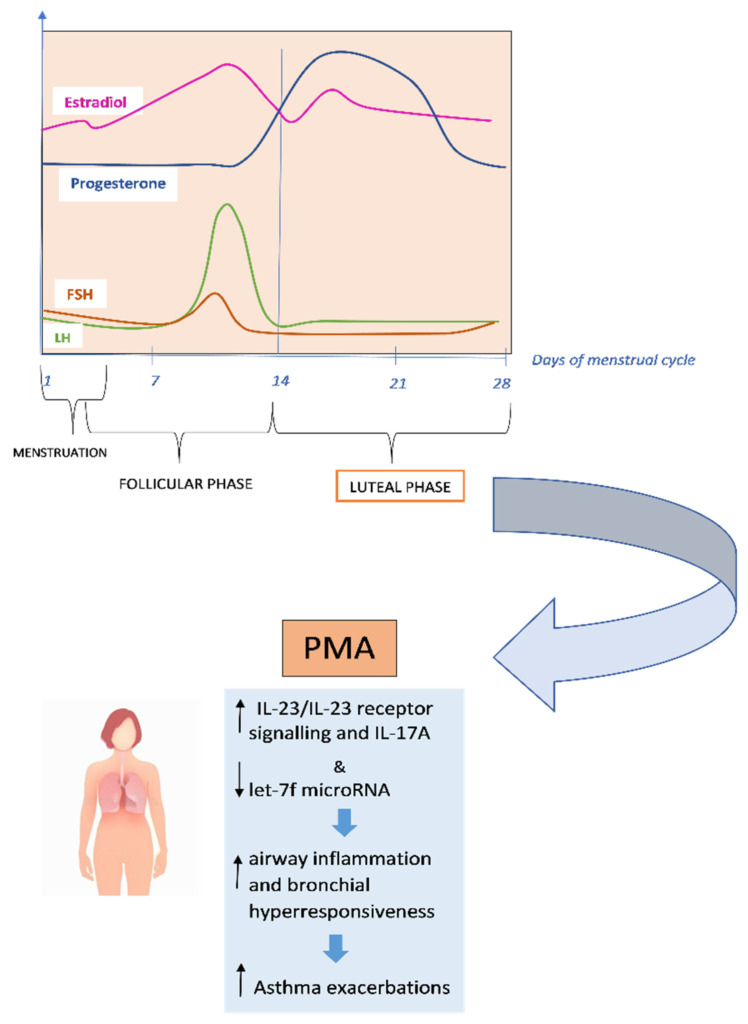
Underlying mechanisms of premenstrual asthma.

Recent researches suggest that premenstrual exacerbations may be caused by increased airway hyperresponsiveness due to impaired β2-adrenoceptor function during the luteal phase of the menstrual cycle [76,79]. It has been found that IL-17A is associated with severe asthma and requires IL-23 receptor (IL-23R) signalling, which is adversely controlled by let-7f microRNA [80] [Figure 2].

In the study by Newcomb et al., researchers found that estrogen and progesterone, in individuals with severe asthma, increased IL-23/IL-23 receptor signalling and IL-17A production while decreased let-7f microRNA expression. Compared to men, TH17 cells of both women with severe asthma and healthy controls produced more IL-17A, increased the expression of IL-23R, while decreased let-7f microRNA expression [81]. In the research by Semik-Orzech et al., thirty-three women of childbearing age were prospectively followed through the measure of sex hormones and the determination of sputum inflammatory cell count and serum inflammatory interleukins, on the 10th and 26th day of each cycle during 12 weeks of observation. Thirteen women were diagnosed with PMA, ten were non-PMA asthmatics (*n* = 10), and ten were healthy controls. The authors found that in PMA patients, the cycle’s luteal phase was associated with increased serum 17β-estradiol levels with concurrent higher sputum concentration of IL-5 and IL-8 and inflammatory cell count [82]. PMA is more common in older, overweight and aspirin-sensitive women. These women also tend to have longer asthmatic episodes and more severe exacerbations. They are more likely to experience premenstrual syndrome, shorter menstrual cycles, more severe menstrual bleeding, dysmenorrhea, and shorter periods [83]. Additionally, compared to women without PMA, women with PMA have higher rates of urgent medical visits, absenteeism, and usage of β2-agonist rescue [84]. PMA does not always respond to traditional asthma therapy. According to GINA (Global Initiative for Asthma) guidelines, contraceptive pills and/or leukotriene receptors, antagonists may be useful (evidence D) in the prevention and treatment of PMA [75,85]. Currently, additional hormonal therapies such as estrogens, progestogens, gonadotropin-releasing hormone (GnRH) analogues and androgens are taken into consideration [5,86,87,88,89].

### 1.4. Air Pollution and Risk of Developing Childhood Asthma

Exposure to particular indoor and outdoor pollutants is a risk factor for asthma outcomes, such as exacerbations, hospitalizations, increased asthmatic symptoms and for the development of asthma in childhood [85,90]. Due to the plasticity and vulnerability of target organs and systems during these developmental years as well as the lengthy maturation period of the respiratory, immunological, and detoxification systems, early life and childhood may constitute crucial exposure windows for asthma development. During this period, a number of processes, including oxidative stress and damage, airway remodeling, inflammatory pathways and immunological responses, and increased respiratory sensitivity to aeroallergens, have been proposed as ways in which air pollution influences the development of asthma [91]. Toll-Like Receptors (TLRs), reactive oxygen species (ROS) sensing pathways, and poly-aromatic hydrocarbon (PAH) sensing pathways, including the aryl hydrocarbon receptor, are just a few of the cellular sensing mechanisms that allow cells to be stimulated by pollutants. These, in turn, trigger intracellular signaling pathways that promote inflammation, such as the NFkB and MAPK pathways. According to Glencross et al., the air pollution can activate TLRs, a type of cellular receptor created to detect pathogen-associated molecular patterns (PAMPs) of potentially infectious microbes as well as other similar adverse signals [92].

One of the major factors in outdoor air pollution is an increase in exposure to traffic-related air pollution (TRAP). Traffic-related air pollution is a combination of vehicle exhausts, secondary pollutants produced in the atmosphere, evaporative emissions from cars, and non-combustion emissions (such as road dust, tire wear). There are growing data on the impact of TRAP and other outdoor pollutants on allergy and asthmatic illnesses. The pollutants that have been implicated are gases including nitrogen dioxide (NO_2_) _O_, nitric oxide (NO), nitrogen oxides (NO_x_), and ozone (O_3_), but also particulate matter which is <2.5 µm (PM_2.5_) and <10 µm (PM_10_) in diameter [91,93]. In both cellular and acellular experimental systems, PM can produce reactive oxygen species, and ambient particulate matter is well recognized to lead to oxidative stress and a decrease in endogenous antioxidants [92]. According to GINA 2022 [85], based on a 2019 study, up to 4 million new cases of pediatric asthma (13% of the global incidence) could be linked to exposure TRAP and asthma risk is increased by exposure to external pollution, such as living near a major road [93,94]. A systematic review of the literature suggests that exposure during childhood to TRAP levels below those indicated by the World Health Organization (PM_2.5_ 10µg/m^3^ annual mean and NO_2_ 40µg/m_3_ annual mean) is associated with increased incidence of asthma and increased risk of sensitization to common allergens [93]. In another recent systematic review and meta-analysis, forty-one studies between 1999 and 2016 were analyzed regarding TRAP exposure and the development of asthma in childhood. Based on the evidence, they concluded that there is an association between TRAP exposure and childhood asthma development with a high degree of consistency among the studies analyzed [94].

Like outdoor pollution, exposure to indoor pollution also plays a key role in the development of asthma and as a factor that could influence asthma symptom control. A recent study has evaluated the impact of air pollution on asthma outcomes. According to the results, indoor and outdoor pollution represent a risk factor for asthma outcomes, such as exacerbations, hospitalizations and increased asthmatic symptoms [90]. Prospective cohort studies are providing even more evidence that exposing children to ambient air pollution increases their chance of acquiring asthma [95].

Many citizens of developed nations spend their time indoors, proving the significance of indoor air quality. Tobacco smoke is one of the most significant indoor pollutions. According to Eguiluz-Gracia et al. alteration of the airway microbiome and direct contact of tobacco smoke against the bronchial epithelium play a key role in the risk of developing asthma and for asthma exacerbations in already asthmatic individuals. Healthy humans have a variety of different bacteria, viruses, and fungi living in their respiratory tracts. This colonization could change in reaction to alterations in the immediate environment, such as exposure to tobacco smoke, possibly resulting in a protracted condition of bacterial dysbiosis. Additionally, tobacco smoke increases the generation of airway mucus, inhibits mucociliary clearance, and promotes low-grade pulmonary inflammation [96]. According to a number of cross-sectional studies and meta-analisys, it would seem that there is a link between electronic cigarettes usage and asthma in teenagers, but further research is required to confirm and better understand this association [97,98].

Nonsmokers who inhale tobacco smoke in a variety of microenvironments, such as homes or workplaces, were in significant health risk (second-hand smoke (SHS) exposure) [99]. Exposure to tobacco smoke in pediatric settings through the secondhand smoking during pregnancy and infancy is linked to the development of asthma, poor asthma management, and more severe asthma in exacerbations children, for this reason according to GINA guidelines after birth or throughout pregnancy, children shouldn’t be exposed to ambient tobacco smoke [85]. The principal indoor pollutants also include other substances like volatile organic compounds (VOC) like formaldehyde, carbon monoxide (CO), and nitrogen dioxide (NO_2_). One of the main sources of indoor NO_2_ emissions is gas-powered heating and cooking equipment. Numerous studies have found a link between indoor NO_2_ and children’s asthma symptoms [100]. The sources of indoor VOCs comes from materials for construction or consumer goods (cleaning agents, cosmetics, air fresheners, building materials, solvents), according to Nurmatov et al. there is only little evidence linking exposure to these VOCs to the development of asthma and AR in both children and adults, as well as to the worsening of asthma and AR symptoms [101]. In another systematic review, according to Maung et al. asthma symptoms have been linked to higher VOC and CO levels [102]. More studies are needed to further understand how VOCs affect the onset/aggravation of childhood asthma.

The management of acceptable air quality will become increasingly difficult due to the rapid global development in urbanization, industrial production, road traffic, but it represents one of the major current challenges. The treatment of asthma exacerbations caused by air pollution is similar to standard clinical practice. Current guidelines recommend that all asthmatic patients use a controlled asthma treatment. Inhaled corticosteroids (ICSs), the first-line treatment for asthma, were found to be effective in reducing adverse reactions to pollutant exposure [85,90]. Numerous risk-reduction strategies have been suggested. These include personal strategies, local and governmental initiatives. Personal strategies, such as driving with the windows closed, maintaining automobile air filtration systems and internal circulation, switching from motorized to non-motorized travel such as cycling and walking, using close-fitting facemasks when air pollution levels are high [90]. Some key actions have already been taken by governments such as replacement of fossil fuels with renewable energy sources and industry commitment to completely phase out coal power. The adoption of the European Union’s (EU) environmental policy framework over the past ten years has significantly decreased emissions of various air pollutants and improved air quality throughout Europe [96,103,104].

## 2. Conclusions

Non-allergic asthma affects a smaller number of pediatric asthma patients than the allergic form does. However, knowing the factors that can trigger asthmatic exacerbations is useful to the pediatrician to detect and prevent symptoms, especially in those patients who do not respond adequately to traditional therapy. The low Th2 expression model is, currently, a rapidly developing promising field for the identification and the treat of solvable clinical problems and for the creation of novel targeted therapy. Some indicated preventive measures such as practicing adequate pre-exercise warming and reducing exposure to cold air during physical activity, reducing exposure to tobacco smoke and air pollution, and better understanding of the mechanisms of virus-induced asthma could be effective in reducing childhood asthma and improving asthma control.

Moreover, a field of great interest concerns the therapeutic interventions for the prevention of infantile and childhood asthma. In the study by Yoshihara et al., an early intervention regimen of initiating sodium cromoglycate inhalation within two years of onset of asthma showed a marked improvement in the long-term prognosis of childhood asthma, especially for children with severe asthma [105]. Similarly, leukotriene receptor antagonists, such as montelukast, have been shown to prevent asthma exacerbations in young children with virus-induced wheezing [106]. In addition, montelukast in mice models has been shown to block and reverse the asthmatic chronic inflammation-induced airway remodeling [107]. In certain Asian nations, asthmatic preventative treatment is started at a younger age—for instance, at six months—than in Western countries [108,109]. However, there are few research on cohorts of such young children, and those that do exist are regionally focused. We think it is more practical to apply non-pharmacological preventive interventions, like those already discussed in the manuscript, and to encourage breastfeeding in newborns, especially in those with a family history of atopy and prone to atopic march.

In recent years, the role of the microbiome has received extensive research. It is known that bronchial tree is characterized by the presence of a microbial flora that differs between diseased and non-diseased people [110]. This is particularly evident in asthmatic subjects, who are characterized by a specific correlation between their lower intestinal and bronchial microbiome expression and their pathologic condition [111]. More and more data support the importance of intestinal microbiome as a protective and barrier tool against infections, immunological tolerance and maintenance of adequate intestinal homeostasis [112]. Since the intestine and bronchial microbiomes grow at the same period of life (before the age of three), it is easy to conceive a cross interaction between the two systems, giving the bronchial microbiome the same or similar importance as the intestinal microbiome. In asthma, more severe airway obstruction and neutrophilic airway inflammation are linked to airway colonization by potentially harmful microbes. This altered colonization may contribute to the emergence of an asthma phenotype that reacts less favorably to current asthma treatments [20,113]. New studies on lung microbiome are expected to shed light on many aspects of severe asthma, as well as on non-Th2-mediated asthma [114].

## Data Availability

Not applicable.

## References

[1] Miraglia del Giudice M., Piacentini G.L., Capasso M., Capristo C., Maiello N., Boner A.L., Capristo A.F. (2007). Formoterol, montelukast, and budesonide in asthmatic children: Effect on lung function and exhaled nitric oxide. Respir. Med..

[2] Di Cicco M.E., Leone M., Scavone M., Del Giudice M.M., Licari A., Duse M., Brambilla I., Ciprandi G., Caffarelli C., Tosca M. (2021). Intermittent and mild persistent asthma: How therapy has changed. Acta Biomed..

[3] Lo Valvo L., Leonardi S., Marseglia G.L., Del Giudice M.M., Salpietro C., Ciprandi G., La Rosa M. (2011). Inhalation therapy in asthmatic and not asthmatic children. Int. J. Immunopathol. Pharmacol..

[4] Licari A., Ciprandi G., Marseglia G.L., Silvestri M., Tosca M.A., Anastasio E., Brambilla I., Caffarelli C., Castagnoli R., Chini L.N. (2020). Asthma in children and adolescents: The ControL’Asma project. Acta Biomed..

[5] Murphy V.E., Gibson P.G. (2009). Premenstrual Asthma: Prevalence, Cycle-to-Cycle Variability and Relationship to Oral Contraceptive Use and Menstrual Symptoms. J. Asthma.

[6] Reddel H.K., Bacharier L.B., Bateman E.D., Brightling C.E., Brusselle G.G., Buhl R., Cruz A.A., Duijts L., Drazen J.M., FitzGerald J.M. (2021). Global Initiative for Asthma Strategy 2021: Executive summary and rationale for key changes. Eur. Respir. J..

[7] Jones T.L., Neville D.M., Chauhan A.J. (2018). Diagnosis and treatment of severe asthma: A phenotype-based approach. Clin. Med..

[8] Indinnimeo L., Chiappini E., Miraglia Del Giudice M., Capristo C., Cardinale F., Cazzato S., Chiamenti G., Chinellato I., Corsello G., Cutrera R. (2018). Guideline on management of the acute asthma attack in children by Italian Society of Pediatrics. Ital. J. Pediatr..

[9] Miraglia del Giudice M., Matera M.G., Capristo C., Conte M., Santaniello F., Chinellato I., Leonardi S., Miraglia del Giudice M.C., Perrone L. (2013). LABAs in asthmatic children: Highlights and new inside. Pulm. Pharmacol. Ther..

[10] Indinnimeo L., Bertuola F., Cutrera R., De Benedictis F.M., Di Pietro P., Duse M., Gianiorio P., Indirli G., La Grutta S., La Rosa M. (2009). Clinical evaluation and treatment of acute asthma exacerbations in children. Int. J. Immunopathol. Pharmacol..

[11] Ciprandi G., Cuppari C., Salpietro A.M., Tosca M.A., Rigoli L., Grasso L., La Rosa M., Marseglia G.L., Del Giudice M.M., Salpietro C. (2012). Serum IL-23 Strongly and Inversely Correlates with FEV1 in Asthmatic Children. Int. Arch. Allergy Immunol..

[12] Decimo F., Capristo C., Amelio R., Maiello N., Capristo A.F., Miraglia Del Giudice M. (2011). Evaluation of bronchial hyperreactivity with mannitol dry powder challenge test in a paediatric population with intermittent allergic asthma or allergic rhinitis. Int. J. Immunopathol. Pharmacol..

[13] Tosca M.A., Schiavetti I., Medone E., del Giudice M.M., Ciprandi G. (2022). Role of FEF25–75 in children sent by primary care paediatricians for asthma diagnosis. Acta Paediatr..

[14] Asero R., Tripodi S., Dondi A., Di Rienzo Businco A., Sfika I., Bianchi A., Candelotti P., Caffarelli C., Povesi Dascola C., Ricci G. (2015). Prevalence and Clinical Relevance of IgE Sensitization to Profilin in Childhood: A Multicenter Study. Int. Arch. Allergy Immunol..

[15] Baos S., Calzada D., Cremades-Jimeno L., Sastre J., Picado C., Quiralte J., Florido F., Lahoz C., Cárdaba B. (2018). Nonallergic Asthma and Its Severity: Biomarkers for Its Discrimination in Peripheral Samples. Front. Immunol..

[16] Del Giudice M.M., Licari A., Brambilla I., Tosca M.A., Ciprandi G. (2020). Allergen Immunotherapy in Pediatric Asthma: A Pragmatic Point of View. Child.

[17] Fahy J.V. (2015). Type 2 inflammation in asthma--present in most, absent in many. Nat. Rev. Immunol..

[18] Peters M.C., Mekonnen Z.K., Yuan S., Bhakta N.R., Woodruff P.G., Fahy J.V. (2014). Measures of gene expression in sputum cells can identify TH2-high and TH2-low subtypes of asthma. J. Allergy Clin. Immunol..

[19] Ciprandi G., Caimmi D., del Giudice M.M., La Rosa M., Salpietro C., Marseglia G.L. (2012). Recent developments in United airways disease. Allergy Asthma Immunol. Res..

[20] Green B.J., Wiriyachaiporn S., Grainge C., Rogers G.B., Kehagia V., Lau R., Carroll M.P., Bruce K.D., Howarth P.H. (2014). Potentially pathogenic airway bacteria and neutrophilic inflammation in treatment resistant severe asthma. PLoS ONE.

[21] Uddin M., Nong G., Ward J., Seumois G., Prince L.R., Wilson S.J., Cornelius V., Dent G., Djukanović R. (2010). Prosurvival activity for airway neutrophils in severe asthma. Thorax.

[22] Bullens D.M.A., Truyen E., Coteur L., Dilissen E., Hellings P.W., Dupont L.J., Ceuppens J.L. (2006). IL-17 mRNA in sputum of asthmatic patients: Linking T cell driven inflammation and granulocytic influx?. Respir. Res..

[23] Raedler D., Ballenberger N., Klucker E., Böck A., Otto R., Prazeres Da Costa O., Holst O., Illig T., Buch T., Von Mutius E. (2015). Identification of novel immune phenotypes for allergic and nonallergic childhood asthma. J. Allergy Clin. Immunol..

[24] Peters S.P. (2014). Asthma phenotypes: Nonallergic (intrinsic) asthma. J. Allergy Clin. Immunol. Pract..

[25] Panettieri R.A. (2016). Neutrophilic and Pauci-immune Phenotypes in Severe Asthma. Immunol. Allergy Clin. N. Am..

[26] Kostakou E., Kaniaris E., Filiou E., Vasileiadis I., Katsaounou P., Tzortzaki E., Koulouris N., Koutsoukou A., Rovina N. (2019). Acute Severe Asthma in Adolescent and Adult Patients: Current Perspectives on Assessment and Management. J. Clin. Med..

[27] Novak N., Bieber T. (2003). Allergic and nonallergic forms of atopic diseases. J. Allergy Clin. Immunol..

[28] Klain A., Indolfi C., Dinardo G., Contieri M., Decimo F., Miraglia Del Giudice M. (2021). Exercise-Induced Bronchoconstriction in Children. Front. Med..

[29] Lin L.L., Huang S.J., Ou L.S., Yao T.C., Tsao K.C., Yeh K.W., Huang J.L. (2019). Exercise-induced bronchoconstriction in children with asthma: An observational cohort study. J. Microbiol. Immunol. Infect..

[30] Brooks E.G., Hayden M.L. (2003). Exercise-induced asthma. Nurs. Clin. North Am..

[31] Minic P.B., Sovtic A.D. (2017). Exercise intolerance and exercise-induced bronchoconstriction in children. Front. Biosci..

[32] Leonardi S., Vitaliti G., Marseglia G.L., Caimmi D., Lionetti E., Miraglia Del Giudice M., Salpietro C., Spicuzza L., Ciprandi G., La Rosa M. (2012). Function of the airway epithelium in asthma. J. Biol. Regul. Homeost. Agents.

[33] Parsons J.P., Hallstrand T.S., Mastronarde J.G., Kaminsky D.A., Rundell K.W., Hull J.H., Storms W.W., Weiler J.M., Cheek F.M., Wilson K.C. (2013). An official American Thoracic Society clinical practice guideline: Exercise-induced bronchoconstriction. Am. J. Respir. Crit. Care Med..

[34] Weiler J.M., Brannan J.D., Randolph C.C., Hallstrand T.S., Parsons J., Silvers W., Storms W., Zeiger J., Bernstein D.I., Blessing-Moore J. (2016). Exercise-induced bronchoconstriction update-2016. J. Allergy Clin. Immunol..

[35] Duong M.L., Subbarao P., Adelroth E., Obminski G., Strinich T., Inman M., Pedersen S., O’Byrne P.M. (2008). Sputum eosinophils and the response of exercise-induced bronchoconstriction to corticosteroid in asthma. Chest.

[36] Hsieh C.C., Goto H., Kobayashi H., Chow W.C., Peng W.H., Tang R.B. (2009). Changes in Serum Eosinophil Cationic Protein Levels after Exercise Challenge in Asthmatic Children. J. Asthma.

[37] Tahan F., Saraymen R., Gumus H. (2008). The role of lipoxin A4 in exercise-induced bronchoconstriction in asthma. J. Asthma.

[38] Hallstrand T.S., Chi E.Y., Singer A.G., Gelb M.H., Henderson W.R. (2007). Secreted phospholipase A2 group X overexpression in asthma and bronchial hyperresponsiveness. Am. J. Respir. Crit. Care Med..

[39] Zietkowski Z., Skiepko R., Tomasiak M.M., Bodzenta-Lukaszyk A. (2007). Endothelin-1 in exhaled breath condensate of allergic asthma patients with exercise-induced bronchoconstriction. Respir. Res..

[40] Parsons J.P., Mastronarde J.G. (2009). Exercise-induced asthma. Curr. Opin. Pulm. Med..

[41] Del Giacco S.R., Firinu D., Bjermer L., Carlsen K.-H. (2015). Exercise and asthma: An overview. Eur. Clin. Respir. J..

[42] Randolph C. (2013). Pediatric exercise-induced bronchoconstriction: Contemporary developments in epidemiology, pathogenesis, presentation, diagnosis, and therapy. Curr. Allergy Asthma Rep..

[43] Dryden D.M., Spooner C.H., Stickland M.K., Vandermeer B., Tjosvold L., Bialy L., Wong K., Rowe B.H. (2010). Exercise-Induced Bronchoconstriction and Asthma. Evid. Rep. Technol. Assess..

[44] Godfrey S., Springer C., Bar-Yishay E., Avital A. (1999). Cut-off points defining normal and asthmatic bronchial reactivity to exercise and inhalation challenges in children and young adults. Eur. Respir. J..

[45] Del Giudice M.M., Pezzulo A., Capristo C., Alterio E., Caggiano S., de Benedictis D., Capristo A.F. (2009). Leukotriene modifiers in the treatment of asthma in children. Ther. Adv. Respir. Dis..

[46] Duse M., Santamaria F., Verga M.C., Bergamini M., Simeone G., Leonardi L., Tezza G., Bianchi A., Capuano A., Cardinale F. (2022). Correction: Inter-society consensus for the use of inhaled corticosteroids in infants, children and adolescents with airway diseases. Ital. J. Pediatr..

[47] Piacentini G.L., Peroni D.G., Miraglia Del Giudice M., Bodini A., Costella S., Vicentini L., Boner A.L. (2002). Effect of montelukast on exhaled NO in asthmatic children exposed to relevant allergens. Pediatr. Allergy Immunol..

[48] Wanrooij V.H.M., Willeboordse M., Dompeling E., Van De Kant K.D.G. (2014). Exercise training in children with asthma: A systematic review. Br. J. Sports Med..

[49] Tancredi G., Quattrucci S., Scalercio F., De Castro G., Zicari A.M., Bonci E., Cingolani S., Indinnimeo L., Midulla F. (2004). 3-min step test and treadmill exercise for evaluating exercise-induced asthma. Eur. Respir. J..

[50] Milanese M., Miraglia del Giudice E., Peroni D.G. (2019). Asthma, exercise and metabolic dysregulation in paediatrics. Allergol. Immunopathol..

[51] Beuther D.A., Martin R.J. (2006). Efficacy of a heat exchanger mask in cold exercise-induced asthma. Chest.

[52] Brenner A.M., Loren M.L., Weiser P.C., Krogh L.A. (1980). Effectiveness of a Portable Face Mask in Attenuating Exercise-Induced Asthma. JAMA.

[53] Lorente F., Laffond E., Moreno E., Dávila I. (2001). Viral infection and asthma: Immunologic mechanisms. Allergol. Immunopathol..

[54] Hansbro N.G., Horvat J.C., Wark P.A., Hansbro P.M. (2008). Understanding the mechanisms of viral induced asthma: New therapeutic directions. Pharmacol. Ther..

[55] Johnston S.L., Pattemore P.K., Sanderson G., Smith S., Lampe F., Josephs L., Symington P., Toole S., Myint S.H., Tyrrell D.A.J. (1995). Community study of role of viral infections in exacerbations of asthma in 9-11 year old children. BMJ.

[56] Yoo J.K., Kim T.S., Hufford M.M., Braciale T.J. (2013). Viral infection of the lung: Host response and sequelae. J. Allergy Clin. Immunol..

[57] Wu P., Hartert T.V. (2011). Evidence for a causal relationship between respiratory syncytial virus infection and asthma. Expert Rev Anti Infect. Ther..

[58] Busse W.W., Lemanske R.F., Gern J.E. (2010). Role of viral respiratory infections in asthma and asthma exacerbations. Lancet.

[59] Jackson D.J., Johnston S.L. (2010). The role of viruses in acute exacerbations of asthma. J. Allergy Clin. Immunol..

[60] Stewart C.J., Hasegawa K., Wong M.C., Ajami N.J., Petrosino J.F., Piedra P.A., Espinola J.A., Tierney C.N., Camargo C.A., Mansbach J.M. (2018). Respiratory Syncytial Virus and Rhinovirus Bronchiolitis Are Associated With Distinct Metabolic Pathways. J. Infect. Dis..

[61] Corne J.M., Marshall C., Smith S., Schreiber J., Sanderson G., Holgate S.T., Johnston S.L. (2002). Frequency, severity, and duration of rhinovirus infections in asthmatic and non-asthmatic individuals: A longitudinal cohort study. Lancet.

[62] Zhu Z., Tang W., Gwaltney J.M., Wu Y., Elias J.A. (1997). Rhinovirus stimulation of interleukin-8 in vivo and in vitro: Role of NF-kappaB. Am. J. Physiol..

[63] Terajima M., Yamaya M., Sekizawa K., Okinaga S., Suzuki T., Yamada N., Nakayama K., Ohrui T., Oshima T., Numazaki Y. (1997). Rhinovirus infection of primary cultures of human tracheal epithelium: Role of ICAM-1 and IL-1beta. Am. J. Physiol..

[64] Message S.D., Laza-Stanca V., Mallia P., Parker H.L., Zhu J., Kebadze T., Contoli M., Sanderson G., Kon O.M., Papi A. (2008). Rhinovirus-induced lower respiratory illness is increased in asthma and related to virus load and Th1/2 cytokine and IL-10 production. Proc. Natl. Acad. Sci. USA.

[65] Legg J.P., Hussain I.R., Warner J.A., Johnston S.L., Warner J.O. (2003). Type 1 and type 2 cytokine imbalance in acute respiratory syncytial virus bronchiolitis. Am. J. Respir. Crit. Care Med..

[66] Román M., Calhoun W.J., Hinton K.L., Avendaño L.F., Simon V., Escobar A.M., Gaggero A., Díaz P.V. (1997). Respiratory syncytial virus infection in infants is associated with predominant Th-2-like response. Am. J. Respir. Crit. Care Med..

[67] Mejías A., Chávez-Bueno S., Jafri H.S., Ramilo O. (2005). Respiratory syncytial virus infections: Old challenges and new opportunities. Pediatr. Infect. Dis. J..

[68] Al-nakib W., Tyrrell D.A.J. (1992). Drugs against rhinoviruses. J. Antimicrob. Chemother..

[69] Edwards M.R., Johnson M.W., Johnston S.L. (2006). Combination therapy: Synergistic suppression of virus-induced chemokines in airway epithelial cells. Am. J. Respir. Cell Mol. Biol..

[70] Skevaki C.L., Christodoulou I., Spyridaki I.S., Tiniakou I., Georgiou V., Xepapadaki P., Kafetzis D.A., Papadopoulos N.G. (2009). Budesonide and formoterol inhibit inflammatory mediator production by bronchial epithelial cells infected with rhinovirus. Clin. Exp. Allergy.

[71] Darveaux J.I., Lemanske R.F. (2014). Infection-related asthma. J. Allergy Clin. Immunol. Pract..

[72] Kumar S., Roy R.D., Sethi G.R., Saigal S.R. (2019). Mycoplasma pneumoniae infection and asthma in children. Trop. Doct..

[73] Liu X., Wang Y., Chen C., Liu K. (2021). Mycoplasma pneumoniae infection and risk of childhood asthma: A systematic review and meta-analysis. Microb. Pathog..

[74] Shah R., Newcomb D.C. (2018). Sex Bias in Asthma Prevalence and Pathogenesis. Front. Immunol..

[75] Fuseini H., Newcomb D.C. (2017). Mechanisms Driving Gender Differences in Asthma. Curr. Allergy Asthma Rep..

[76] Matteis M., Polverino F., Spaziano G., Roviezzo F., Santoriello C., Sullo N., Bucci M.R., Rossi F., Polverino M., Owen C.A. (2014). Effects of sex hormones on bronchial reactivity during the menstrual cycle. BMC Pulm. Med..

[77] Yung J.A., Fuseini H., Newcomb D.C. (2018). Hormones, sex, and asthma. Ann. Allergy Asthma Immunol..

[78] Bernstein J.A. (2020). Progestogen Sensitization: A Unique Female Presentation of Anaphylaxis. Curr. Allergy Asthma Reports.

[79] Tan K.S. (2001). Premenstrual asthma: Epidemiology, pathogenesis and treatment. Drugs.

[80] Fuseini H., Yung J.A., Cephus J.Y., Zhang J., Goleniewska K., Polosukhin V.V., Peebles R.S., Newcomb D.C. (2018). Testosterone Decreases House Dust Mite-Induced Type 2 and IL-17A-Mediated Airway Inflammation. J. Immunol..

[81] Newcomb D.C., Cephus J.Y., Boswell M.G., Fahrenholz J.M., Langley E.W., Feldman A.S., Zhou W., Dulek D.E., Goleniewska K., Woodward K.B. (2015). Estrogen and progesterone decrease let-7f microRNA expression and increase IL-23/IL-23 receptor signaling and IL-17A production in patients with severe asthma. J. Allergy Clin. Immunol..

[82] Semik-Orzech A., Skoczyński S., Pierzchała W. (2017). Serum estradiol concentration, estradiol-to-progesterone ratio and sputum IL-5 and IL-8 concentrations are increased in luteal phase of the menstrual cycle in perimenstrual asthma patients. Eur. Ann. Allergy Clin. Immunol..

[83] Sánchez-Ramos J.L., Pereira-Vega A.R., Alvarado-Gómez F., Maldonado-Pérez J.A., Svanes C., Gómez-Real F. (2017). Risk factors for premenstrual asthma: A systematic review and meta-analysis. Expert Rev. Respir. Med..

[84] Thornton J., Lewis J., Lebrun C.M., Licskai C.J. (2012). Clinical characteristics of women with menstrual-linked asthma. Respir. Med..

[85] 2022 GINA Main Report—Global Initiative for Asthma—GINA. https://ginasthma.org/gina-reports/.

[86] Tan K.S., McFarlane L.C., Lipworth B.J. (1997). Modulation of airway reactivity and peak flow variability in asthmatics receiving the oral contraceptive pill. Am. J. Respir. Crit. Care Med..

[87] Vélez-Ortega A.C., Temprano J., Reneer M.C., Ellis G.I., McCool A., Gardner T., Khosravi M., Marti F. (2013). Enhanced generation of suppressor T cells in patients with asthma taking oral contraceptives. J. Asthma.

[88] Calcaterra V., Nappi R.E., Farolfi A., Tiranini L., Rossi V., Regalbuto C., Zuccotti G. (2022). Perimenstrual Asthma in Adolescents: A Shared Condition in Pediatric and Gynecological Endocrinology. Child.

[89] Pereira-Vega A., Sánchez Ramos J.L., Maldonado Pérez J.A., Vázquez Oliva R., Bravo Nieto J.M., Vázquez Rico I., Ignacio García J.M., Romero Palacios P., Alwakil Olbah M., Medina Gallardo J.F. (2012). Premenstrual asthma and leukotriene variations in the menstrual cycle. Allergol. Immunopathol..

[90] Tiotiu A.I., Novakova P., Nedeva D., Chong-Neto H.J., Novakova S., Steiropoulos P., Kowal K. (2020). Impact of Air Pollution on Asthma Outcomes. Int. J. Environ. Res. Public. Health.

[91] Gowers A.M., Cullinan P., Ayres J.G., Anderson H.R., Strachan D.P., Holgate S.T., Mills I.C., Maynard R.L. (2012). Does outdoor air pollution induce new cases of asthma? Biological plausibility and evidence; a review. Respirology.

[92] Glencross D.A., Ho T.R., Camiña N., Hawrylowicz C.M., Pfeffer P.E. (2020). Air pollution and its effects on the immune system. Free Radic. Biol Med..

[93] Bowatte G., Lodge C., Lowe A.J., Erbas B., Perret J., Abramson M.J., Matheson M., Dharmage S.C. (2015). The influence of childhood traffic-related air pollution exposure on asthma, allergy and sensitization: A systematic review and a meta-analysis of birth cohort studies. Allergy.

[94] Khreis H., Kelly C., Tate J., Parslow R., Lucas K., Nieuwenhuijsen M. (2017). Exposure to traffic-related air pollution and risk of development of childhood asthma: A systematic review and meta-analysis. Environ Int..

[95] Gehring U., Wijga A.H., Koppelman G.H., Vonk J.M., Smit H.A., Brunekreef B. (2020). Air pollution and the development of asthma from birth until young adulthood. Eur. Respir. J..

[96] Eguiluz-Gracia I., Mathioudakis A.G., Bartel S., Vijverberg S.J.H., Fuertes E., Comberiati P., Cai Y.S., Tomazic P.V., Diamant Z., Vestbo J. (2020). The need for clean air: The way air pollution and climate change affect allergic rhinitis and asthma. Allergy.

[97] Di Cicco M., Sepich M., Ragazzo V., Peroni D.G., Comberiati P. (2020). Potential effects of E-cigarettes and vaping on pediatric asthma. Minerva Pediatr..

[98] Xian S., Chen Y. (2021). E-cigarette users are associated with asthma disease: A meta-analysis. Clin. Respir. J..

[99] Drago G., Perrino C., Canepari S., Ruggieri S., L’Abbate L., Longo V., Colombo P., Frasca D., Balzan M., Cuttitta G. (2018). Relationship between domestic smoking and metals and rare earth elements concentration in indoor PM 2.5. Environ. Res..

[100] Gillespie-Bennett J., Pierse N., Wickens K., Crane J., Howden-Chapman P., Shields H., Viggers H., Free S., Phipps R., Fjallstrom P. (2011). The respiratory health effects of nitrogen dioxide in children with asthma. Eur. Respir. J..

[101] Nurmatov U.B., Tagiyeva N., Semple S., Devereux G., Sheikh A. (2015). Volatile organic compounds and risk of asthma and allergy: A systematic review. Eur. Respir. Rev..

[102] Maung T.Z., Bishop J.E., Holt E., Turner A.M., Pfrang C. (2022). Indoor Air Pollution and the Health of Vulnerable Groups: A Systematic Review Focused on Particulate Matter (PM), Volatile Organic Compounds (VOCs) and Their Effects on Children and People with Pre-Existing Lung Disease. Int. J. Environ. Res. Public Health.

[103] Annesi-Maesano I. (2017). The air of Europe: Where are we going?. Eur. Respir. Rev..

[104] EUR-Lex-32016L2284-EN EUR-Lex. https://eur-lex.europa.eu/legal-content/EN/TXT/?uri=uriserv:OJ.L_2016.344.01.0001.01.ENG&toc=OJ:L:2016:344:TOC.

[105] Yoshihara S., Kanno N., Yamada Y., Ono M., Fukuda N., Numata M., Abe T., Arisaka O. (2006). Effects of early intervention with inhaled sodium cromoglycate in childhood asthma. Lung.

[106] Fitzgerald D.A., Mellis C.M. (2006). Leukotriene receptor antagonists in virus-induced wheezing: Evidence to date. Treat. Respir. Med..

[107] Debelleix S., Siao-Him Fa V., Begueret H., Berger P., Kamaev A., Ousova O., Marthan R., Fayon M. (2018). Montelukast reverses airway remodeling in actively sensitized young mice. Pediatr. Pulmonol..

[108] Guan R., Liu Y., Ren D., Li J., Xu T., Hu H. (2020). The efficacy and safety of fluticasone propionate/formoterol compared with fluticasone propionate/salmeterol in treating pediatric asthma: A systematic review and meta-analysis. J. Int. Med. Res..

[109] Kahlon G.K., Pooni P.A., Bhat D., Dhooria G.S., Bhargava S., Arora K., Gill K.S. (2021). Role of montelukast in multitrigger wheezers attending chest clinic in Punjab, India. Pediatr. Pulmonol..

[110] Hilty M., Burke C., Pedro H., Cardenas P., Bush A., Bossley C., Davies J., Ervine A., Poulter L., Pachter L. (2010). Disordered microbial communities in asthmatic airways. PLoS ONE.

[111] García-Rivero J.L. (2020). The Microbiome and Asthma. Arch. Bronconeumol..

[112] Lozupone C.A., Stombaugh J.I., Gordon J.I., Jansson J.K., Knight R. (2012). Diversity, stability and resilience of the human gut microbiota. Nature.

[113] Di Cicco M., Pistello M., Jacinto T., Ragazzo V., Piras M., Freer G., Pifferi M., Perpni D. (2018). Does lung microbiome play a causal or casual role in asthma?. Pediatr Pulmonol..

[114] Huang Y.J. (2013). Asthma microbiome studies and the potential for new therapeutic strategies. Curr. Allergy Asthma Rep..

